# Strengths and challenges of a school-based sexual and reproductive health program for adolescents in Chile

**DOI:** 10.1371/journal.pone.0265309

**Published:** 2022-03-23

**Authors:** Alexandra Obach, Michelle Sadler, Báltica Cabieses, Pascale Bussenius, Patricia Muñoz, Claudia Pérez, Carla Urrutia

**Affiliations:** 1 Programa de Estudios Sociales en Salud, Instituto de Ciencias e Innovación en Medicina, Facultad de Medicina Clínica Alemana, Universidad del Desarrollo, Santiago, Chile; 2 Departamento de Historia y Ciencias Sociales, Facultad de Artes Liberales, Universidad Adolfo Ibáñez, Santiago, Chile; 3 Corporación Municipal de Lo Prado, Ilustre Municipalidad de Lo Prado, Santiago, Chile; National Research Centre of Egypt, EGYPT

## Abstract

**Background:**

Adolescent sexual and reproductive health services in Chile have been primarily provided through health centers. Although some school-based initiatives have been implemented, to date, these have not been assessed. This study aims to identify strengths and challenges of the affectivity and sexuality component of the school-based 3A Program, a health program which seeks to prevent risk behaviors and promote healthy lifestyle habits within public schools (addressing health topics which in Spanish begin with the letter ‘A’, hence ‘3A’), implemented in the municipality of Lo Prado, city of Santiago.

**Methods:**

We carried out a qualitative study with a descriptive-interpretative approach in three schools. We conducted in-depth interviews with students, teachers, health professionals, and school principals (N = 44); and focus groups with students (N = 3), teachers and health personnel (N = 3). The interviews were analyzed using thematic analysis.

**Results:**

Participants highlight the integrative approach to health and to sexual and reproductive health promoted in the 3A Program, which is enhanced by the collaboration of interdisciplinary health teams. Permanent and expedited student access to sexual and reproductive health care is achieved, and affectional bonds are developed between students and the Program’s health staff. The Program assists female participants to imagine and form identities that are not inherently tied to motherhood. It also assists boys and LGBTQ+ adolescents in feeling included as relevant actors in sexual and reproductive health and decision making. The delivery of contraception in schools is highly valued. The most significant challenge identified is ensuring effective and ongoing collaboration between health staff and teachers.

**Conclusion:**

Participants value the effectivity and sexuality component of the 3A Program as an initiative to improve adolescents’ access to sexual and reproductive health care. Our findings suggest that this Program could be replicated throughout the region and the country to improve the quality and accessibility of health services for adolescents.

## Introduction

The Sustainable Development Goals (SDGs) and the Global Strategy for Women’s, Children’s and Adolescents’ Health consider adolescent health as a key factor for the social, economic and political progress of all countries [1–3]. Despite this recognition and international commitments to improve friendly health care [4], adolescents experience significant health needs, and generally require improved access to health services [5]. In Latin America, indicators for the adolescent/young population regarding sexual and reproductive health (SRH) are alarming: progress in the reduction of HIV/AIDS is slower than in other regions of the world [6, 7]; the rate of teenage pregnancy (15–19 years) is the second highest in the world [8, 9]; a poor quality of sexual education programs and limited access to contraceptive methods and safe abortions is common to many countries [10–12].

The development of intersectoral strategies is a promising strategy for strengthening adolescent health, especially between the health and educational sectors [13–16]. As the evidence demonstrates, school-based health programs are easily accessible, may be the first point of contact for adolescents seeking health services, and they may increase healthcare access for low-income adolescents who often experience inequities in health care access and outcomes [5]. SRH programs in schools have been reported to increase contraceptive use and reduce unplanned pregnancy and sexually transmitted infections (STIs) [17–19]. Such programs are scarce in Latin America, and there is limited evidence supporting their impact [20]. Initiatives such as the CERCA Program and the Andean Plan to Prevent Teen Pregnancy (PLANEA), which have been run in several countries of the Region, have shown that establishing strong bonds between young people and their teachers, health professionals, parents and friends can contribute to effective SRH programs [9, 21]. Despite these initiatives, an integral approach to the health of adolescents and young people in Latin America is long overdue [9, 20].

In Chile, following the World Health Organization’s and Pan American Health Organization´s guidelines for the implementation of Adolescent Friendly Health Services [22, 23], and within the framework of PLANEA [9], the National Adolescent and Youth Health Program was created in 2009 [24]. Since then, it has been implementing youth-friendly health services for adolescents ages 10–19, especially in the area of SRH, with a notable success in decreasing the adolescent birth rate: in 2018, Chile recorded Latin America’s lowest rate, estimated at 23 births per 1,000 girls aged 15–19 [25]. However, this rate obscures significant inequalities: in 2017, the difference between the highest and lowest income deciles in teenage pregnancies among 15–19 years old was 63 times (i.e. below 0.2% in the richest decile, and above 11% in the poorest) [25]. Furthermore, this age group has high rates of STIs including HIV [26].

Several studies in Chile have identified the barriers adolescents experience when attempting to access SRH services. Such services are primarily provided through health centers, with challenges such as lack of privacy and confidentiality, and excessively bureaucratic admission processes [27–31]. The country’s SRH framework has been described as being primarily focused on the biological aspects of health, reinforcing a risk approach, with healthcare focusing mainly on heterosexual women and the prevention of pregnancy and STIs [27, 29, 30]. Young men and the LGBTQ+ young population have understandably expressed their dissatisfaction with the health care system [29, 31]. Adolescents have evaluated sexual education as the worst aspect of educational establishments in the last three National Youth Surveys carried out between 2012 and 2018 [32].

Although Youth-Friendly Health Services’ protocols include joint actions for the health and education sectors, there is no detail on how to implement them, and therefore actions vary considerably across different contexts [24]. There are very few experiences of school-based health programs delivered directly in schools, of which the most promising initiative is the 3A Program (3A) which has been carried out since 2011 in the Lo Prado municipality (Chile’s smallest administrative subdivision) of the city of Santiago [33]. This municipality is a low-income segregated neighborhood located in the Western area of the city, characterized by high levels of poverty, teenage pregnancy and social vulnerability [34].

Given the lack of studies regarding the impact of school-based health programs in Latin America and Chile, our study aims to examine the strengths and challenges of the 3A school-based health program, and more specifically, its affectivity and sexuality component (detailed below). To our knowledge, this is the first study in the country to describe such a program. We hope that the study will contribute to discussions regarding progress and innovation in adolescent health access in the region and country.

## Methods

### Study design and context

We conducted a qualitative study with a descriptive-interpretative approach, seeking to understand how people give meaning to their social environment and how they interpret it [35]. The study followed a case-study method, which enables an in-depth exploration of phenomena in its naturally occurring context, involving the study of a bounded system (or case) within a contemporary setting through detailed, in-depth data collection [36].

The study case is the 3A Program, a joint initiative of the municipality of Lo Prado’s health and education offices, which seeks to prevent risk behaviors and promote healthy lifestyle habits within public schools. Three main pillars frame this goal: affectivity and sexuality; self-care; and nutrition and physical activity (in Spanish, these concepts begin with the letter ‘A’, hence ‘3A’). The program is funded by the municipality’s education and health budgets. This funding enables the employment of midwives, social workers, psychologists and nutritionists. In each school, a health room provides a space where professionals provide direct care and counseling to students, in addition to workshops in classrooms.

The study focused on the affectivity and sexuality component of the Program. This component includes workshops on affective relationships, decision-making in sexuality, sexual and reproductive rights, contraceptive methods and STIs. It also encompasses SRH care and counseling; coordination with the management teams of the schools; counselling with parents and participation in parents’ meetings. Contraception is also available in the school setting (with the exception of interventions that require a clinical setting, such as long acting reversible contraception, which is provided by appointments in primary health centers). All of these actions have been incorporated into the schools’ curriculum.

In Lo Prado municipality, 12 public schools run the 3A Program. Three of these include the high school years (in Chile, the four last years of school). Among these three, there were 30 classes and 808 students attending high school during the fieldwork period. The students at these three establishments present similarly highly vulnerable socio-demographic characteristics. The schools were included in the study because the majority of the affectivity and sexuality component of the Program is implemented during high school.

### Sample and recruitment

Fieldwork was carried out between March and September of 2018 by three experienced researchers in qualitative methodologies (authors AO, MS and PB). Four categories of respondents were recruited: school principals, head teachers, health staff working in the affectivity and sexuality component of the 3A Program, and students. The inclusion criteria for participants was: to be school principals, head teacher of high school classes, and health staff working in the affectivity and sexuality component of the 3A Program. For students, the inclusion criteria was to be in high school with the only exclusion criteria of not speaking Spanish fluently (as some foreign students, especially from Haiti, did not).

The principal investigator (AO) first presented the study to each of the three school principals, who agreed to participate in the study. With their collaboration, all head teachers and 3A health staff were contacted to take part in meetings where the research team presented the study and invited them to participate in in-depth interviews and/or focus groups. Of the 30 head teachers, 21 agreed to collaborate. All health staff working in the affectivity and sexuality component of the 3A Program in these schools agreed to participate: nine social workers, four midwives and three phycologists. In one school, one nutritionist also approached the study team, and was included as she collaborated frequently with the Program.

From the total of 30 classes, 12 were selected to invite students, corresponding to one class of each grade per school, with a total of 325 students. In each of these classes, teachers and 3A health staff presented the study and those students who wished to participate completed a contact information form. 63 expressed their interest in participating, after which the research team contacted them to arrange contact by telephone, email, WhatsApp and/or Instagram. 38 students ultimately took part in the study (others did not reply to follow-up contacts or did not attend scheduled appointments). The recruitment of students could have presented selection bias: on one hand, it was carried out with the support of 3A health staff and school teachers, which could have influenced who participated; on the other, as students were invited to participate voluntarily, those who were more involved in the 3A Program could have been over-represented.

As shown in Table 1, of the 38 participating students, 21 were women and 17 men. Regarding age groups, 13 were 14–16 years old, while 25 were 17–19. Regarding their nationality, 26 were Chilean, 8 Colombian, 2 Peruvian, 1 Venezuelan and 1 Haitian. In relation to sexual orientation, 29 declared to be heterosexual, 5 bisexual, and 4 homosexual. Of all students, 2 had been parents and 1 was pregnant. Of the 17 health staff who participated in the study, 15 were women and 2 men; 7 were aged 23–30, 6 were 31–40, and 4 were 41 years or older. Regarding their professions, 9 were social workers, 4 were midwives, 3 were psychologists and 1 was a nutritionist. 6 of them had been working in the 3A Program for 1–2 years, 8 for 3–4 years, and 3 for 5 or more years. 21 teachers and 3 school principals took part in the study. Of these, 7 were women and 17 men; 3 were 23–30 years old, 5 were 31–40, and 16 were 41 or older; 9 had been working in the schools for 1–2 years, 6 for 3–4 years and 9 for 5 or more years.

**Table 1:** Characteristics of participants in in depth-interviews and focus groups(N = 79).

**Students (N = 38)**		**N**	**(%)** *
Gender	Female	21	55
	Male	17	45
Age	14–16	13	34
	17–19	25	66
Nationality	Chilean	26	68
	Colombian	8	21
	Peruvian	2	5
	Venezuelan	1	3
	Haitian	1	3
Sexual orientation	Heterosexual	29	76
	Bisexual	5	13
	Homosexual	4	11
Parenthood	No children	35	92
	One child	2	5
	Pregnant	1	3
**3A health staff (N = 17)**		**N**	**(%)**
Sex	Female	15	88
	Male	2	12
Profession	Social worker	9	53
	Midwife	4	24
	Psychologist	3	18
	Nutritionist	1	6
Age	23–30	7	41
	31–40	6	35
	41-more	4	24
Years working in the 3A Program	1–2	6	35
	3–4	8	47
	5-more	3	18
**Teachers and school principals (N = 24)**		**N**	**(%)**
Sex	Female	7	29
	Male	17	71
Age	23–30	3	13
	31–40	5	21
	41-more	16	67
Years working in the school	1–2	9	38
	3–4	6	25
	5-more	9	38

*Percentages may not add exactly to 100% as they were rounded to whole numbers.

### Data collection

In-depth interviews and focus groups were used to collect information because they achieve different objectives. Interviews enable more detailed, in-depth information at an individual level, while focus groups explore points of encounter and disagreement at the group level [36]. Interview guides were built to ensure consistency across in-depth interviews and focus groups, based on research objectives. The following topics were covered: (i) implementation of 3A in schools; (ii) strengths and challenges of the affectivity and sexuality component of the Program; (iii) for health staff, main challenges faced when working in schools; (iv) for teachers, integration of health professionals and their work on SRH health issues with students; (v) for students, experiences with the Program and with its health staff; (vi) other new topics related to the study that participants wished to discuss. The questions were asked with an open approach which allowed participants to expand freely on their perceptions and experiences. Researchers kept a research journal where they took field notes during and after encounters. In-depth interviews and focus groups were carried out in the schools, in rooms with adequate privacy, during the school day or immediately after. Researchers/authors OB, MS and PB moderated all interviews and focus groups. In-depth interviews were moderated by one researcher, and focus groups by two researchers/authors, in order to guarantee homogeneity across encounters. No one else was present in the encounters other than recruited participants and researchers.

#### In-depth interviews

A total of 44 in-depth interviews were carried out: 3 with school principals, 13 with 3A health staff, 10 with school teachers, and 18 with students. With the exception of school principals, interviews were carried out within each category of participant (3A health staff, teachers and students) until data saturation was achieved. The duration of the interviews was 40–65 minutes.

#### Focus groups

6 focus groups were run: 3 with 3A health staff and teachers (one per school) and 3 with students (one per school). In each school, 3A health staff and teachers who had expressed an interest in taking part in the study, and had not participated in an in-depth interview, were invited to a focus-group (4 health staff and 11 teachers). Additionally, some participants who had already been interviewed expressed their interest in joining the groups, which was considered a contribution to the debate (8 health staff and 5 teachers). In sum, 16 teachers and 12 health professionals took part in the three focus groups, each of which contained 9 or 10 participants. The 20 students who were interested in participating and had not been interviewed, were invited to take part in a focus group. One focus group with male and female students was conducted in each school: two had 7 participants and one had 6.

Two researchers were present in each focus group; one as moderator, the second taking detailed notes on the group’s dynamics and environment, to supplement and guide interpretation of the transcripts. The duration of the focus groups was 75–90 minutes.

### Analysis

In-depth interviews and focus groups were audio recorded and transcribed verbatim using Microsoft Word, and each transcript was checked for accuracy against the original recording by the researcher who had moderated the encounter. The transcripts were analysed using thematic analysis, a qualitative method that enables thematic patterns to be identified from the collected data [36], with the support of software QSR NVivo 11. Three researchers (AO, MS and PB) conducted the analysis, based on the following steps: familiarization of the transcripts by reading and re-reading the texts; identification of preliminary codes, by noting emerging issues from the data; grouping related codes in clusters; creating a code book with main and subordinate codes; identifying patterns across interviews. During each of these steps, the researchers compared notes and resolved disagreements by discussion consensus was reached. Then, all other authors (BC, PM, CP, CU) were involved in interpreting the coded data in order to reach conclusions.

The verbatim quotations selected for this article were translated from Spanish by a native English-speaker translator and checked by authors to verify the translations had captured their original meaning.

In order to assess the rigor of the study, triangulation of responses from several techniques (in-depth interviews and focus groups) and participants (principals, 3A health staff, teachers and students) was carried out [37].

### Ethical considerations

The project was approved by the Ethics Committee of Universidad del Desarrollo (IRB 2018–06), which is officially registered by the US Office for Human Research Protections. Participants received information about the objectives and procedures of the study orally and in writing. All adults signed an informed consent. Participants who were 17 years or younger signed an informed assent and a signed consent was also obtained from their parents and/or legal tutors. Confidentiality and anonymity were ensured by replacing names with interview codes.

## Results

The strengths and challenges of the affectivity and sexuality component of the 3A Program are presented, integrating findings from the three schools studied, as participants from the three establishments shared common appreciations about the Program.

### 1. Strengths of the 3A program

#### 1.1. Integrative health and SRH approach

*Integrative and multidisciplinary approach to health*. Participants of all groups -principals, teachers, 3A health staff and students- agreed that one of the main strengths of the Program is its comprehensive approach to health, an acknowledgment that all dimensions of health are interdependent. The students interviewed appreciate that the Program addresses ‘meaningful issues of life’, beyond obtaining good grades and passing courses, as is illustrated in the following quotation:

“Unlike other schools, here we have more support. It´s much more than learning what you are supposed to learn in school which is maths, history; they teach you more important things for life; to take care of yourself, protect yourself. They address meaningful issues of life beyond the typical school contents.” (Student, interview N°17)

According to all respondents, the use of interdisciplinary health teams -social workers, psychologists, midwives and nutritionists- has been key in achieving an integrative approach. Social workers act as support in situations of social vulnerability, and work alongside psychologists to help students cope. According to students, expedited access to psychologists is crucial in addressing common personal issues such as low self-esteem. Adolescents believe that the 3A staff provide them with coping tools, as one student articulated:

“I had a kind of couple therapy with the psychologist, because we were having problems with my boyfriend. She talked to both of us and that helped my relationship to flow. She also helped me cope with the problems I was having at home, with all that was happening… I value that very much.” (Student, interview N°13)

Midwives are also recognized by students, especially girls, as fundamental in addressing their health needs. Several students acknowledge their surprise when finding out midwives could also promote an integrative approach to health, beyond the mere delivery of contraceptive methods, as detailed below.

*Holistic view of sexuality*. All categories of participants agree that, for adolescents, SRH is typically addressed from a risk perspective, with a focus on avoiding or reducing sexual activity in order to avoid early pregnancy or STIs. A midwife claims that the Program: “Is much more than giving a condom and showing a PowerPoint on the horrors of STIs, as it is still done in many school contexts” (3A health staff, interview N°30). This study reveals that the prevention of STIs and of pregnancy are important topics, but should be addressed within a preventive framework which enhances health, promotes self-care and the care of others. As one student reveals:

“I have understood what STIs are, […] and now I understand the importance of using a condom. Even if the person has never had sexual intercourse, they still need to have condoms, just in case, you never know… they have encouraged a lot that we should take care of ourselves and of our partners.” (Student, interview N°9)

According to participants, the affectivity and sexuality component of the Program encourages self-care and responsibility in sexual and emotional relationships. Effective dialogue, communicating one’s feelings, and caring for the feeling of others, are a focus of the Program. 3A also encourages reflection on gender stereotypes and norms regarding sexuality. The concept of ‘machismo’ and its negative health consequences is one example. The Program also raises awareness about the naturalization of violence in many adolescent relationships, and helps students to recognize this harmful attitude. As one student explains:

“I have friends whose boyfriends are violent; so they teach you and they guide you and you can distinguish what is good, what not to do and what you shouldn’t allow to happen.” (Student, interview N°11)

According to interviewees, the reflection on gender roles promoted by 3A health staff is also important when talking about consent and coercion in sexual relationships, as articulated by one student:

“When men forced women to have sex, that is not pleasant for the woman… if the woman tells you no, it is no, you cannot force her to have intercourse.” (Student, interview N°3)

Additionally, students from different nationalities note that the Program has helped them to recognize how gender roles and norms are culturally embedded and expressed in diverse ways.

*The importance of pleasure in sexuality*. Students highlight that the affectivity and sexuality workshops address pleasure as a fundamental component of sexuality and as a sexual right, giving them tools to explore their bodies and identify their erogenous zones, which is illustrated in the following quotations: “I think it’s super good that they teach you the types of orgasms and how you can experience them” (student, interview N°9); “We have talked in class about the G-spot, and that each person can have it in different parts” (student, interview N°6).

Students also describe how the Program emphasizes that sex should be pleasurable for both men and women, and that communication is crucial in order to have healthy sexual relations. The following quotes from students illustrate these points:

“We discuss that in a full relationship with your partner, the idea is that what you feel is mutual, that not only one person enjoys.” (Student, interview N°11)

“About pleasure, they have told us that it has to be mutual, that you have to like it, if you don’t like it, you have to talk to your partner, that you have to talk to each other about these things.” (Student, interview N°6).

*Life project beyond motherhood for adolescent girls*. Teachers and 3A health staff attest that the Program provides tools for adolescent girls to develop a life project, reversing cultural mandates of motherhood as a goal. In the words of a social worker, “We help them work on their confidence, to focus on goals other than motherhood and believe they can achieve them; it is one of our main goals as a Program” (3A health staff, interview N°20). A midwife complements this idea:

“Our idea is to broaden their perspectives on what their life project can look like, beyond becoming mothers at an early age; to give them tools to believe they can achieve other purposes” (3A health staff, interview N°23).

#### 1.2. Continuous access to SRH care

*SRH care within adolescents’ daily environments*. Respondents unanimously agreed on the value of having permanent and expedited student access to SRH counseling and care, free from the bureaucratic barriers found in primary health care centers. In their opinion, SRH care in schools considerably reduces challenges such as a lack of confidentiality, and the shame that occurs in health centers, as adolescents can be recognized by neighbors. This is illustrated by students in the following quotations:

“A friend went to get condoms at the health center and the pharmacist was a neighbor of his family, so everybody found out. Instead, here you feel sure that they will not tell anyone.” (Student, interview N°4).

“The advantage here [in school] is that you come directly in the [health] room and they take care of you. Instead, going to the health center makes you feel ashamed; you have to get a number, they shout out your name and everybody is listening.” (Student, interview N°2).

3A staff report that female adolescents most frequently attend the health rooms, mainly for contraception, which is linked to the awareness that the students have acquired about the risk of pregnancy and self-care in sexuality:

“We go to the midwife often, to talk about how to take care of ourselves… there have been pregnant girls in my class and most of us go for that reason, we don’t want it happening to us.” (Student, interview N°17)

*Health rooms as safe spaces*. Students consider health rooms safe places to speak about anything with health staff. In terms of its accessibility, students value the fact that the health room is permanently open to whoever wants to attend, without having to make an appointment. As a student notes, “It is open to all public whenever you need, and you will almost always find somebody to talk” (student, interview N°8). They value that the health room has the necessary equipment to take care of their health consults, such as stretchers, chairs, scales, pressure tapping equipment; but mostly that they are cozy places to be. In addition to medical equipment, there are books and art materials which make students feel welcome, enhancing the feeling of a nurturing and safe meeting space for students and staff. A student illustrates this:

“It’s cool, it’s warm in the room when it’s cold outside, and there are always kind and pleasant people to talk to (…). It’s very cool, in that room there are never bad faces, the truth is that they are always happy.” (Student, interview N°5).

Health rooms are not the only space where the 3A health staff develop the Program, as they also walk around schools, using public spaces such as hallways, yards and dining rooms to engage in informal conversations with adolescents. As a midwife describes, this encourages those adolescents who do not yet feel confident to attend the health rooms, to start conversations about their health needs.

*Improved access of adolescent men to SRH care*. Participants appreciate that the Program is also available to male adolescents seeking SRH services. A midwife comments on this integral approach to sexuality:

“Men hardly go [to the health center] (…) they are ashamed to be called out loud by the midwife in front of others, but here in school they feel more confident and comfortable talking about sexuality.” (3A health staff, interview N°29)

Some boys report having learned through the Program, that they are active subjects in SRH care, and that they should be responsible for the prevention of unwanted pregnancies and STIs:

“It is always assumed that women have to take care of themselves [referring to contraception], but now I see that this is something good for my health too and that I also have be responsible.” (Student, interview N°4).

*Improved access for LGBTQ+ adolescents to SRH care*. Participants claim that 3A incorporates an explicit approach towards gender equity and social equality, including the topics of diversity and sexual dissidence. A student addresses this point, saying:

“We have had a lot of discussions about sexual diversity and they do handle it well. They have explained to us what gender identity is and the difference between gender, sex, identity and all those things.” (Student, interview N°14).

Students note that understanding the concepts of sex, gender, sexual identity, sexual orientation and diversity, have helped to reduce bullying and have improved school relationships in general:

“I came to this school in the first year of high school, when at home nobody knew that I was gay. They suspected, but I think they didn’t take it seriously and my grandmother, she is super old-fashioned, very closed-minded, so I suffered a lot because I didn’t know how she would react. So I was going to talk to the *tías* [‘aunts’, referring to the 3A staff], I told them that I didn’t like lying to my mother and they helped me to tell my family little by little.” (Student, interview N°12).

3A staff participating in the study highlight the promotion of acceptance, empathy and non-discriminatory treatment for adolescents of diverse sexual identities. This is reflected in the learnings described by students attending workshops, such as the awareness of homophobic violence and discrimination. As one adolescent explains, “What I value the most is that they are teaching us that people should not be discriminated against for any reason” (Student, interview N°1). When needed, 3A staff supports students through their sexual identity development. One of the interviewed midwives described how she supported a transgender adolescent throughout her transition process.

*Access to condoms and contraceptive methods in schools*. Another of the most valued aspects of the Program is expedited access in schools to condoms and contraceptive methods, as illustrated in the following quotes:

“They encourage us to prevent [pregnancy and STIs] and give us many condoms, they are always telling us to be careful, to take care of ourselves.” (Student, interview N°8)

“A person who wants to have sex will go to the midwife, ask for condoms, the midwife will give a little talk, but will not ask very intimate things.” (Student, interview N°17).

The delivery of contraception, including condoms, is accompanied by midwife counseling. Students highlight that boys and girls are provided the same information, which includes heterosexual and homosexual practices:

“Before, only men were taught how to use the condom, but now women are also taught; they bring condoms and a wooden thing [dildo] and they make everyone practice.” (Student, interview N°15)

“They do not only teach you about heterosexual relationships, they also teach you how to use a condom when you have oral sex with a man and a woman, which is super good.” (Student, interview N°7).

Participants acknowledge that the Program has had a visible impact, reducing the rate of teenage pregnancies in the three schools studied. One student states:

“There are less pregnancies because it’s much easier to prevent it; you have all the information and you can go to the midwife and she gives you pills right away.” (Student, interview N°2)

#### 1.3. Affectional bond between 3A staff and students

Participants agree that the development of affectionate bonds between adolescents and 3A staff is a fundamental aspect of the Program. These bonds support pregnancy and STI prevention, early interventions in SRH and the promotion of healthy sexuality. This is possible thanks to the physical closeness and continuous presence of health staff in schools, which allows for ongoing interactions and promotes trust. One student states that, “Here there is confidence; they ask you, they worry; I did not expect that” (Student, interview N°2). Students also emphasize that they do not feel judged or discriminated against by the health staff in schools: “They love us as we are” (Student, interview N°7). Likewise, adolescents feel that the 3A staff are welcoming and warm, even beyond office hours:

“They are super good people. They will never tell you ‘no’, they could ask you to wait a bit but never a ‘no’ for an answer. They may be having lunch, and you come crying, and they leave their lunch and go to talk to you. I am closest with the psychologist and with the midwife, with whom I have spoken the most. I also have a relationship with the social worker but it is because she is super nice, that’s why, and because she sometimes teaches us too.” (Student, interview N°8).

Additionally, students value the way that 3A staff take the time to know them, recognize and enhance their talents and potential, and help them to develop their self-esteem:

“The funny thing about the 3A *tías* is that they know that we are good at something, and they look for ways to make us improve. For example, I was having problems with my grades, and the *tía* suggested I could make drawings, or do creative things, which I really enjoy. She took the time to think about what I am good at and what I can improve on.” (Student, interview N°7)

### 2. Challenges of the 3A program

#### 2.1. Tensions between teachers and 3A staff

According to the 3A health staff, one of the main obstacles to implementing the Program in schools is resistance from teaching staff. Before the Program, teachers attended to most of the students’ needs, including sexual education and emotional support. Although the presence of the 3A personnel reduces teacher workloads, some teachers are reluctant to lose their bond with their students. As one student describes:

“The teachers can be mean to the 3A staff (…). For example, there’s a girl having a nervous crisis in class; the psychologist needs to get her out and the teachers won’t let her: ‘She is in my class, you are not allowed to take her out.’ It’s much better to miss a class and to get help than to be cutting your arms, or trying to hang yourself, or crying all day (…). Some teachers minimize the 3A, they don’t want to see how important it has been for us; it has really helped us a lot.” (Student, interview N°17)

This tension is illustrated in some of the teacher´s comments about health staff: “They call students from the classroom whenever they want, regardless of the educational consequences it could have” (teacher, interview N°39); “They walk a lot around the school in their white aprons, but they do little real work” (teacher, interview N°35).

There are also tensions related to the high expectations that teachers impose on the Program. Health personnel believe that teachers tend to expect immediate changes in the behavior of students. When these expectations are not met, they tend to invalidate and belittle the work done. As one 3A psychologist reports, “There is a misconception of change in the kids from their teachers. I realize that many teachers expect big changes, they come to me and say: ‘But I referred the kid to you a month ago!’” (3A health staff, interview N°25).

According to participants, tensions between health and education staff are due to a lack of true collaboration, as the two communities are not used to working together. A midwife states, “It’s strange for a midwife to be in a school, sometimes I even ask myself: ‘What am I doing in a teachers’ meeting?’ And teachers make sarcastic comments to express their discomfort with us” (3A health staff, interview N°23).

The most significant resistance originates from teachers who have been working in the schools for the longest; newer and younger teachers are more supportive of the Program.

#### 2.2. Rotation of health personnel

As one of the Program’s strengths is in the bond between students and 3A staff, a high turnover of health personnel is a significant challenge. A female student notes:

“Last year there was a woman psychologist and I really liked talking to her; it makes me sad that she left (…). It should not happen because you take time to build trust with a psychologist, you might not have the confidence to talk about your problems with a new person.” (Student, interview N°10)

The rotation of health personnel prompts some students to abandon their healthcare. Clearly, continuity of care is important in the support of adolescents.

#### 2.3. Limited participation of boys and LGBTQ+ adolescents

Although the 3A facilitates access to SRH care for boys and LGBTQ+ adolescents, 3A staff report that their participation could be improved. They note the importance of developing methodologies to motivate males to access the SRH services available to them. A midwife says, “It is not easy to motivate boys, they are ashamed of coming to the midwife, and we are lacking specific tools to approach boys and sexually diverse young people.” (3A health staff, interview N°29)

#### 2.4. Quality of classroom workshops

Improvements could also be made in terms of the quality of the classroom workshops carried out by the 3A staff. Students note that content is repeated each year. A student states, “I have had the 3A Program for several years, and every year they are reinforcing the topics, but always in a similar way” (student, interview N°14). 3A staff acknowledge the issue, and complain that they lack the tools and training required for improved classroom activities.

#### 2.5. Parental resistance

Some parents are also resistant to the Program. They express their concerns that the Program could prompt an earlier start to sexual activity and are concerned about discussions related to diverse gender and sexual identities. A midwife summarises, “Some families do not accept that their kids are having sexual intercourse or that they are exploring their gender identity” (3A health staff, focus group N°2).

## Discussion

Evidence has shown that school-based interventions in SRH and mental health have the potential to improve the availability of services, particularly for young people who are normally underserved [16]. Additionally, health services in schools have the potential to reduce transport costs, increase accessibility and provide links between schools and communities [16]. This is consistent with the results of our study, which illustrates the strengths of the affectivity and sexuality component of the 3A school-based Program, which solves many of the traditional barriers to accessing SRH care in adolescents in Chile. These barriers include challenges related to access to health centers such as physical distance, bureaucratic barriers and lack of confidentiality [27, 29, 30].

According to studies on sexual education and HIV prevention in Latin America, it is crucial that the connection between adolescents and health services is strengthened [9, 20, 38]. Our results show that the 3A Program follows this aim: adolescents experience a closer relationship with health services and staff, as they are integrated into the daily context of school life. This model is particularly relevant in a country such as Chile, where 12-year education is compulsory, and dropouts are low [39]. Health center care is strongly associated with the exercise of authoritative knowledge by health staff, and endorses hierarchical relationships in which adolescents feel patronized and stigmatized [29]. The perception of “closeness” which adolescents report when school health rooms are implemented also speaks to the trust bond that is created with the health staff. These results support the findings of Tabong et al. in Ghana, which highlight the importance of allocating time for school-based SRH services, and the allocation of enough time for interaction between health service providers and students [40].

Studies suggest that many school-based health programs are restricted in providing hormonal birth control [17, 18] and condoms in schools [5]. Other studies show that these programs often emphasize abstinence and do not help adolescents evaluate the risk associated with sexual behaviors [38, 41]. Findings from the Ghana study (quoted above), demonstrate that the majority of respondents -teachers, parents and students- support adolescents’ access to comprehensive SRH information and services, but generally disapprove of the provision of contraception in schools due to concerns that this could promote sexual activity [40]. Unlike the restrictions on access to contraception in various school-based health programs reported in the literature, the 3A Program offers expedited access to contraception and STI prevention methods for adolescents in schools. In all categories of respondents, this is reported as a significant strength of the Program. Additionally, 3A addresses pleasure as a sexual right for adolescents. The results of our study align with studies that highlight that adolescents want to receive accurate and comprehensive SRH information and services in schools [11, 29].

Participants of the 3A Program value its comprehensive approach to health, where sexuality and reproduction are integrated dimensions in a broader conception of health (including affectivity, social relationships, mental health, nutrition, etc.). Thus, 3A challenges traditional reductionist, biological and risk approach perspectives related to adolescents. This integrative approach of health in general, and SRH in particular, is evident in the staffing of the Program, which comprises interdisciplinary teams with midwives, psychologists and social workers.

Despite the strengths of the Program, there are also challenges. Some teaching staff are resistant to the model, and there are additional challenges in regards to effective collaboration between health and teaching staff. This could be addressed by involving teachers in the design and implementation process of programs, thus, making them an active part of its results and successes.

Also, there are challenges regarding the inclusion of the health needs of boys and LGBTQ+ adolescents. Although improved SRH care access has been achieved for these groups, there is more work needed to ensure uptake of SRH health services is sufficient to meet this group’s particular health needs. There is also opportunity to make SRH services even more inclusive. These challenges apply not only to the 3A Program and Chile, but are part of the challenges that adolescent health services face worldwide [11, 23].

Finally, the results show that some parents are resistant to the 3A Program, expressing their concern that both the content of the workshops and the delivery of contraception could induce an early start to sexual activity. This has been also reported in other contexts in the world, with widespread parental resistance to SRH access for students in schools [5].

As part of efforts to promote successful health initiatives, this article shows the positive impact of 3A, as well as opportunities for improvement. The implementation of strategies such as those carried out by 3A are in line with what the evidence raises. 3A contributes to progress towards Sustainable Development Goals (SDG) linked to improving the quality of life and the development of children and adolescents. Additionally, the Program aligns with WHO’s evidence-based interventions proposed for the promotion of adolescent health [WHO Global Acceleration of Measures to Promote Adolescent Health guide, 42].

A limitation of the study is that it presents the results of an SRH intervention program in three schools in a specific district of Chile, hence the findings cannot be generalized to the entire high-school population in Chile. However, the Lo Prado population has similar sociodemographic characteristics to many vulnerable districts in the country. Therefore, the findings are considered useful for broader contexts and the experience can be replicated and extended into other parts of Chile, with adaptation for local contexts.

This is the first study to describe the strengths and challenges of a school-based SRH program for adolescents in Chile, and pursues the ultimate aim of contributing to the development of comprehensive public policies for the promotion of SRH in adolescents. This research could inform administrators, health and school managers and policymakers in SRH for adolescents and young people in Chile and countries which face similar barriers to health care access. Increased knowledge of innovative strategies, especially in countries with low development of such initiatives (such as Chile) is a fundamental step in strengthening SRH services and rights in these age groups, especially in those who are most vulnerable. The ultimate goal in developing and implementing such programs, is progress towards fairer and more inclusive societies.

## Conclusions

The findings of our study show that the 3A Program in general, and specifically its affectivity and sexuality component, are highly valued by participants of the three schools studied, as an initiative to improve and strengthen adolescents’ access to SRH care. The Program’s comprehensive and multidisciplinary approach to health, set in the daily living contexts of adolescents, and the affectional bond developed between students and health staff are its most significant strengths. This study offers opportunity to consider the replicability of 3A in other parts of Chile and in other countries. Acknowledging the strengths and challenges of this Program offers opportunity to develop SRH strategies and programs to provide more just, equitable and relevant healthcare for the adolescent population.

Our findings prompt some recommendations that could serve to strengthen school-based SRH programs, including the following: integrating guidelines for school-based SRH programs in adolescent health policies; involving local authorities in prioritizing these programs in their districts; building technical support for the implementation of SRH programs at municipality and school levels; actively involving teaching staff in the design and implementation processes; encouraging community participation in the programs by involving adolescents, their families and community agents; and monitoring and evaluating existing experiences.

## Supporting information

S1 File Interview guide. (PDF)

## References

[1] Maddaleno M, Morello P, Infante-Espínola F. Health and development of adolescents and young adults in Latin America and the Caribbean: challenges for the next decade. Salud Pública Méx. 2003;45(Suppl 1):S132–S139. 12602156

[2] United Nations. The 2030 Agenda and the Sustainable Development Goals: an opportunity for Latin America and the Caribbean (LC/G.2681-P/Rev.3). Santiago: United Nations; 2018. Available from: https://repositorio.cepal.org/handle/11362/40156

[3] WHO. Global Strategy for Women’s, Children’s and Adolescents’ Health (2016–2030). Survive, thrive, transform. United Nations; 2015. Available from: https://www.who.int/life-course/partners/global-strategy/globalstrategyreport2016-2030-lowres.pdf

[4] WHO. Making health services adolescent friendly: developing national quality standards for adolescent friendly health services. Geneva: World Health Organization; 2012. Available from: chrome-extension://efaidnbmnnnibpcajpcglclefindmkaj/viewer.html?pdfurl=https%3A%2F%2Fapps.who.int%2Firis%2Fbitstream%2Fhandle%2F10665%2F75217%2F9789241503594_eng.pdf&clen=2947791&chunk=true

[5] Moore MJ, Barr E, Wilson K, Griner S. Support for offering sexual health services through school-based health clinics. J Sch Health. 2016;86:660–668. doi: 10.1111/josh.12421 27492935

[6] ONUSIDA. Avanzando hacia las metas 2020: progreso en América Latina y el Caribe. ONUSIDA; 2020. Available from: http://onusidalac.org/1/images/advancing-towards-2020esp-032020.pdf

[7] Khalifa A, Stover J, Mahy M, Idele P, Porth T, Lwamba C. Demographic change and HIV epidemic projections to 2050 for adolescents and young people aged 15–24. Glob Health Action. 2019;12(1):1662685. doi: 10.1080/16549716.2019.1662685 31510887 PMC6746261

[8] PAHO. Accelerating progress toward the reduction of adolescent pregnancy in Latin America and the Caribbean. Report of a technical consultation. Washington, DC: PAHO; 2016. Available from: https://iris.paho.org/handle/10665.2/34493

[9] Córdova Pozo K, Venkatraman CM, Decat P, Nelson N, De Meyer S, Jaruseviciene L, et al. Improving adolescent sexual and reproductive health in Latin America: reflections from an International Congress. Reproductive Health. 2015;12:11. doi: 10.1186/1742-4755-12-11 25616439 PMC4320614

[10] Morgan LM, Roberts E. Reproductive governance in Latin America. Anthropology & Medicine. 2012;19(2):241–254. doi: 10.1080/13648470.2012.675046 22889430

[11] Obach A, Sadler M, Jofré N. Sexual and reproductive health of adolescents in Chile: the role of sexual education. Rev Salud Pública. 2017;19(6):848–854. doi: 10.15446/rsap.V19n6.70023 30183841

[12] INJUV-DESUC. Evidencia a través de la vivencia: una nueva mirada en Chile sobre embarazo adolescente. Santiago: INJUV-DESUC; 2020. Available from: https://sociologia.uc.cl/wp-content/uploads/2020/09/libro-embarazo-adolescente-final.pdf.

[13] WHO. Global school health initiatives: achieving health and education outcomes. Report of a meeting, Bangkok, Thailand, 23–25 November 2015. Geneva: World Health Organization; 2017. Available from: https://www.who.int/healthpromotion/publications/global-school-health-initiatives-report-meeting-2015/en/

[14] WHO, UNESCO, UNICEF, The World Bank. Focusing resources on effective school health: a FRESH start to enhancing the quality and equity of education. World Education Forum; 2001. Available from https://unesdoc.unesco.org/ark:/48223/pf0000124086

[15] Levinson J, Kohl K, Baltag V, Ross DA. Investigating the effectiveness of school health services delivered by a health provider: A systematic review of systematic reviews. PLOS ONE. 2019;14(6):e0212603. doi: 10.1371/journal.pone.0212603 31188826 PMC6561551

[16] Mason-Jones A, Crisp C, Momberg M, Koech J, De Koker P, Mathews C. A systematic review of the role of school-based healthcare in adolescent sexual, reproductive, and mental health. Systematic Reviews. 2012;1:49. doi: 10.1186/2046-4053-1-49 23098138 PMC3621403

[17] Smith P, Novello G, Chacko M. Does immediate access to birth control help prevent pregnancy? A comparison of onsite provision versus off campus referral for contraception at two school-based clinics. Journal of Applied Research on Children: Informing Policy for Children at Risk. 2011;2(2): Article 8. Available from: https://digitalcommons.library.tmc.edu/childrenatrisk/vol2/iss2/8

[18] Minguez M, Santelli J, Gibson E, Orr M, Samant S. Reproductive health impact of a school health center. J of Adolescent Health. 2015;56(3):338–344. doi: 10.1016/j.jadohealth.2014.10.269 25703321

[19] Nair MKC, Paul M, Leena ML, Thankachi Y, Geoge B, Russel PS, Vijayan Pillai H. Effectiveness of a reproductive sexual health education package among school going adolescents. Indian J Pediatr. 2012 Jan; 79(Suppl 1):S64–S68. doi: 10.1007/s12098-011-0433-x 21617909

[20] Caffe S, Plesons M, Camacho AV, Brumana L, Abdool SN, Huaynoca S, et al. Looking back and moving forward: can we accelerate progress on adolescent pregnancy in the Americas? Reprod Health. 2017;14(1):83. doi: 10.1186/s12978-017-0345-y 28705166 PMC5512880

[21] Decat P, Nelson E, De Meyer S, Jaruseviciene L, Orozco M, Segura Z, et al. Community embedded reproductive health interventions for adolescents in Latin America: development and evaluation of a complex multi-centre intervention. BMC Public Health. 2013;13:31. doi: 10.1186/1471-2458-13-31 23311647 PMC3599131

[22] Ministerio de Salud Chile. Servicios de salud integrales, amigables y de calidad para adolescentes. Orientación técnica para la atención primaria de salud. Ministerio de Salud de Chile, 2018. Available from: chrome-extension://efaidnbmnnnibpcajpcglclefindmkaj/viewer.html?pdfurl=https%3A%2F%2Fdiprece.minsal.cl%2Fwp-content%2Fuploads%2F2019%2F03%2F2019.03.04_SS-AMIGABLES-PARA-ADOLESCENTES.pdf&clen=4276816&chunk=true

[23] Mazur A, Brindis C, Decker M. Assessing youth-friendly sexual and reproductive health services: a systematic review. BMC Health Services Research. 2018;18:216. doi: 10.1186/s12913-018-2982-4 29587727 PMC5870922

[24] Ministerio de Salud Chile. Programa Nacional de Salud Integral de Adolescentes y Jóvenes, Plan de acción 2012–2020. Santiago: Ministerio de Salud, Gobierno de Chile de Chile; 2012 Available from: https://www.minsal.cl/portal/url/item/d263acb5826c2826e04001016401271e.pdf

[25] Rodríguez Vignoli J, Roberts Pozo A. El descenso de la fecundidad adolescente en Chile: antecedentes, magnitud, determinantes y desigualdades. Instituto Nacional de la Juventud; 2020.

[26] Cáceres-Burton K. Report: Epidemiological situation of sexually transmitted infections in Chile, 2017. Rev Chil Infectol. 2019;36(2):221–233. doi: 10.4067/S0716-1018201900020022127. 10.4067/S0716-10182019000200221 31344158

[27] Macintyre AK, Montero A, Sagbakken M. From disease to desire, pleasure to the pill: a qualitative study of adolescent learning about sexual health and sexuality in Chile. BMC Public Health. 2015;15:945. doi: 10.1186/s12889-015-2253-9 26399632 PMC4580294

[28] Arenas L, Duran J, Dides C, Fernández C. Educación sexual. En: Dides C, Fernández C(eds.). Salud sexual y salud reproductiva y derechos humanos en Chile, estado de la situación 2016. Santiago: Miles Chile; 2016. Available from: http://www.mileschile.cl/documentos/Informe_DDSSRR_2016_Miles.pdf

[29] Sadler M, Obach A, Luengo X, Biggs A. Estudio barreras de acceso a los servicios de salud para la prevención del embarazo adolescente en Chile. Santiago, Chile: CulturaSalud/Ministerio de Salud; 2011. Available from: http://www.minsal.cl/portal/url/item/ace74d077631463de04001011e011b94.pdf

[30] Rojas G, Eguiguren P, Matalama MI, Palma I, Gálvez T. Adolescents’ access to contraception: perceptions of health workers in Huechuraba, Chile. Rev Panam Salud Pública. 2017;41:e77. doi: 10.26633/RPSP.2017.77 28614485 PMC6645252

[31] Obach A, Sadler M, Aguayo F, Bernales M. Sexual and reproductive health in young men in Chile: results of a qualitative study. Rev Panam Salud Pública. 2018;42:e124. doi: 10.26633/RPSP.2018.124 31093152 PMC6386039

[32] Instituto Nacional de la Juventud. Novena Encuesta Nacional de la Juventud 2018, informe general de resultados. Santiago, Chile: Instituto Nacional de la Juventud; 2019. Available from: http://www.injuv.gob.cl/storage/docs/IX_ENCUESTA_NACIONAL_DE_LA_JUVENTUD_2018.pdf

[33] Obach A, Sadler M, Cabieses B. Intersectoral strategies between health and education for preventing adolescent pregnancy in Chile: findings from a qualitative study. Health Expectations. 2019;22:183–192. doi: 10.1111/hex.12840 30369026 PMC6433321

[34] Prado Lo. Reportes estadísticos 2020 [cited 12 October 2020]. Available from: https://www.bcn.cl/siit/reportescomunales/comunas_v.html?anno=2020&idcom=13117.

[35] Patton M Qualitative research & evaluation methods. Thousand Oaks, CA: SAGE Publications; 2002.

[36] Creswell J. Research design: qualitative, quantitative, and mixed methods approaches. Thousand Oaks, CA: SAGE Publications; 2014.

[37] Cypress B. Rigor or reliability and validity in qualitative research. Dimensions of Critical Care Nursing. 2017;36(4):253–263. doi: 10.1097/DCC.0000000000000253 28570380

[38] DeMaria LM, Galárraga O, Campero L, Walker DM. Educación sobre sexualidad y prevención del VIH: un diagnóstico para América Latina y el Caribe. Rev Panam Salud Publica. 2009;26(6):485–93. doi: 10.1590/s1020-49892009001200003 20107702 PMC4720267

[39] Espinoza Díaz O, Castillo Guajardo D, González LE, Loyola Campos J, Santa Cruz Grau E. School dropout in Chile: a study case in relation to intra-school factors. Educación y Educadores. 2014;17(1):32–50.

[40] Tabong PTN, Maya ET, Adda-Balinia T, Kusi-Appouh D, Birungi H, Tabsoba P, et al. Acceptability and stakeholders perspectives on feasibility of using trained psychologists and health workers to deliver school-based sexual and reproductive health services to adolescents in urban Accra, Ghana. Reproductive Health. 2018;15:122. doi: 10.1186/s12978-018-0564-x 29976216 PMC6034281

[41] Lloyd C. 2007. The role of schools in promoting sexual and reproductive health among adolescents in developing countries. Poverty, gender, and youth Working Paper no. 6. New York: Population Council.

[42] WHO. Global accelerated action for the health of adolescents (AA-HA!): guidance to support country implementation. World Health Organization 2017 WHO/FWC/MCA/17.05. Available from: https://apps.who.int/iris/handle/10665/255418.

