# Supplementary material for: Strengths and challenges of a school-based sexual and reproductive health program for adolescents in Chile
Source: PLoS One. 2022 Mar 23;17(3):e0265309. doi: 10.1371/journal.pone.0265309 (PMC8942266; doi:10.1371/journal.pone.0265309)
Supplement: S1 File — (PDF) [file pone.0265309.s001.pdf]

INTERVIEW GUIDE
Authorities, 3A health staff and school teachers

STUDY FOR THE PROMOTION OF SEXUAL / REPRODUCTIVE HEALTH AND THE PREVENTION OF ADOLESCENT PREGNANCY IN CHILE: IDENTIFYING CHALLENGES AND PROPOSING SOLUTIONS.

Principal Investigator: Alexandra Obach, PhD

I. General questions about the implementation of the 3A Program in the Lo Prado municipality:

1. Could you please describe the implementation of the 3A Program in the Lo Prado municipality?
2. Could you please describe the implementation of the 3A Program in the schools of the municipality?
3. Could you please describe the implementation process of the 3A Program in the high schools of the municipality?
4. How does the 3A Program operate on a daily basis at this school? (Description of the daily routine of the Program and its activities)
5. Which have been the main barriers in the implementation of this Program?
6. Which have been the main facilitators in the implementation of this Program?
7. Which have been the main challenges in the implementation of this Program?
8. How would you describe health rooms?
9. How have these rooms been received by the school community? (managers, teachers, students)

II. Affectivity and Sexuality Component:

10. What are the distinctive characteristics of the affectivity and sexuality component of the 3A Program according to your perception?
11. What activities does this component include?
12. Which of these activities are in the classroom?
13. Which of these activities are outside the classroom?
14. What are the main strengths of this component according to your perception?
15. What are the main weaknesses of this component according to your perception?

III. Exclusive questions for 3A health staff:

16. How is sexual and reproductive health care for adolescents that is delivered in Health Centers different from that delivered in schools through the 3A Program?
17. What are the main challenges for health professionals when working in schools?
18. How do you think adolescents perceive the presence of health professionals in schools?
19. How do you think teachers perceive the presence of health professionals in schools?
20. What differences do you perceive in the relationship established between health professionals and adolescents in the contexts of the Health Center and at school?
21. What are the main barriers for health professionals when working in schools?
22. What are the main strengths that the work in schools entails?
23. Do you identify resistances from teachers regarding the presence of health staff in school?
24. Are there joint teaching activities between teachers and health staff? Which?
25. How has this experience been? (of carrying out joint activities)

26. What has the health staff contributed to the school teachers? (Deepen in relation to the affectivity and sexuality component)
27. What have the teachers contributed to the health staff?
28. What do you think has been the impact of having health staff in school for the students' sexual and reproductive health?
29. What do you think has been the impact of having health staff in the school on adolescent pregnancy?

IV. Exclusive questions for teachers:
30. How have you perceived the work of health professionals in the school?
31. What changes has the work of health professionals meant in the dynamics of the school?
32. How do you think adolescents perceive the work of health staff in schools?
33. How do you think teachers perceive the work of health staff in schools?
34. What have been the greatest benefits of the work of health professionals in schools for the students?
35. What have been the greatest benefits of the work of health professionals in schools for teachers?
36. What have been the greatest difficulties in the work of health professionals in schools for students?
37. What have been the greatest difficulties in the work of health professionals in schools for teachers?
38. Do you identify resistances from teachers regarding the presence of health staff in school?
39. Are there joint teaching activities between teachers and health staff? Which?
40. How has this experience been? (of carrying out joint activities)
41. What has the health staff contributed to the school teachers? (Deepen in relation to the affectivity and sexuality component)
42. What have the teachers contributed to the health staff?
43. What do you think has been the impact of having health staff in school for the students' sexual and reproductive health?
44. What do you think has been the impact of having health staff in the school on adolescent pregnancy?

Facultad de Medicina
Clínica Alemana - Universidad del Desarrollo

INTERVIEW GUIDE
Students

STUDY FOR THE PROMOTION OF SEXUAL / REPRODUCTIVE HEALTH AND THE PREVENTION OF ADOLESCENT PREGNANCY IN CHILE: IDENTIFYING CHALLENGES AND PROPOSING SOLUTIONS.
Principal Investigator: Alexandra Obach, PhD

I. General questions about the implementation of the 3A Program in the Lo Prado municipality:

1. Do you know the 3A Program?
2. How would you describe it?
3. In which 3A Program activities have you participated?
4. What did you think of those activities?
5. What aspects of the 3A Program do you value the most?
6. What aspects of the 3A Program do you value the least?
7. What has the 3A Program given you? (Investigate areas of knowledge, areas of socialization, emotional areas, mental health)
8. What do you consider to be the most innovative aspects of this Program?
9. How would you describe health rooms?
10. Do you use these rooms? Reasons why yes/no. What have you used them for? When/ in which schedules have you used them?
11. What is your opinion of the affectivity and sexuality component of the 3A Program?
12. What activities does this component include?
13. What have you learned about this topic through the Program?
14. What would you improve about the Program and the affectivity and sexuality component specifically?

II. Exclusive questions about health staff:

15. Do you know the Friendly Spaces of the Health Center in your community? Have you gone?
16. How is sexual and reproductive health care for adolescents that is delivered in Health Centers different from that delivered in schools through the 3A Program?
17. How has your experience of having health professionals available in the school been?
18. Does this make a difference from other schools?
19. How is the relationship of the students with the health staff?
20. What kind of relationship do they establish?
21. For what reasons do students look for health staff?
22. Are health professionals accessible within the school?
23. Do you think health professionals know about sexuality issues that interest you?
24. Are health teams available to discuss sexuality issues with students?
25. What do you think of teenage pregnancy?
26. Does having health staff at school help prevent it? How?
27. What do you think of STIs?
28. Does having health staff at school help prevent them? How?
29. Do health professionals who are in schools handle issues of sexual diversity?
30. What are the strengths of having health professionals at school?
31. What are the weaknesses of having health teams at school?

VII. Exclusive questions about teachers:

32. What information do teachers handle on sexual and reproductive health?
33. How is sexual education taught by teachers at school?
34. Are teachers available to discuss sexuality issues with students?
35. What kind of bond do students have with teachers?
36. Is the bond between students and teachers and health staff different? If so, how/ in what ways?
37. Are teachers and health staff aligned in their work at school?
38. Do you identify resistances from teachers regarding the presence of health staff in school?
39. Are there joint teaching activities between teachers and health staff? Which?
40. How has this experience been? (of carrying out joint activities)
41. What has the health staff contributed to the school teachers? (Deepen in relation to the affectivity and sexuality component)
42. What have the teachers contributed to the health staff?
43. What do you think has been the impact of having health staff in school for the students' sexual and reproductive health?
44. What do you think has been the impact of having health staff in the school on adolescent pregnancy?

Facultad de Medicina
Clínica Alemana - Universidad del Desarrollo

GUÍA DE ENTREVISTA
Autoridades, Equipo de Salud y Educadores

ESTUDIO ETNOGRÁFICO PARA LA PROMOCIÓN DE LA SALUD SEXUAL/REPRODUCTIVA Y LA PREVENCIÓN DEL EMBARAZO ADOLESCENTE EN CHILE: IDENTIFICANDO DESAFÍOS Y PROPONIENDO SOLUCIONES.

Investigadora Principal: Alexandra Obach, PhD

I. Preguntas generales acerca de la implementación del Programa 3A en la comuna de Lo Prado:

1. ¿Cómo ha sido la trayectoria de implementación del Programa 3A en la comuna de Lo Prado?
2. ¿Cómo ha sido la trayectoria de implementación del Programa 3A en los colegios de la comuna?
3. ¿Cómo ha sido la trayectoria de implementación del Programa 3A en los colegios de enseñanza media de la comuna?
4. ¿Cómo opera el programa 3A cotidianamente en este colegio? Descripción del cotidiano del programa y sus actividades.
5. ¿Cuáles han sido las principales barreras en la implementación de este Programa?
6. ¿Cuáles han sido los principales facilitadores en la implementación de este Programa?
7. ¿Cuáles han sido los principales desafíos en la implementación de este Programa?
8. ¿Cómo describiría las salas de salud o salas de bienestar?
9. ¿Cómo han sido recepcionadas estas salas por parte de la comunidad escolar? (directivos, profesores, alumnos/as)

II. Componente Afectividad y Sexualidad:

10. ¿Cuáles son las características distintivas del componente efectividad y sexualidad del Programa 3A de acuerdo a su percepción?
11. ¿Qué actividades contempla este componente?
12. ¿Cuáles de dichas actividades son en aula?
13. ¿Cuáles de dichas actividades son fuera de aula?
14. ¿Cuáles son las principales fortalezas de este componente de acuerdo a su percepción?
15. ¿Cuáles son las principales debilidades de este componente de acuerdo a su percepción?

III. Preguntas exclusivas para equipos de salud:

16. ¿En qué se diferencia la atención de salud sexual y reproductiva a adolescentes y jóvenes que se entrega en los centros de salud y la que se entrega en el colegio a través del Programa 3A?
17. ¿Cuáles son los principales desafíos para los profesionales de la salud de insertarse dentro de los colegios?
18. ¿Cómo cree usted que perciben los(as) adolescentes la presencia de los equipos de salud en los colegio?
19. ¿Cómo cree usted que perciben los(as) profesores(as) la presencia de los equipos de salud en los colegio?
20. ¿Qué diferencias percibe en la relación que se establece entre profesional de la salud y adolescentes en el contexto centro de salud y en el colegio?
21. ¿Cuáles son las principales barreras para los equipos de salud en dicha inserción?
22. ¿Cuáles son las principales fortalezas que conlleva dicha inserción?
23. ¿Identifica resistencias por parte de profesores(as) en la entrada de los equipos de salud al colegio?

24. ¿Existen actividades docentes conjuntas entre profesores(as) y equipos de salud? ¿Cuáles?
25. ¿Cómo ha sido esta experiencia?
26. ¿Qué han aportado los equipos de salud a los profesores(as) del colegio? Profundizar en lo relativo al componente afectividad y sexualidad.
27. ¿Qué han aportado los profesores(as) a los equipos de salud?
28. ¿Cuál cree que ha sido el impacto en la salud sexual y reproductiva de los(as) alumnos la instalación de los equipos de salud en el colegio?
29. ¿Cuál cree que ha sido el impacto en el embarazo adolescente la instalación de los equipos de salud en el colegio?

IV. Preguntas exclusivas para profesores:

30. ¿Cómo ha percibido la entrada de equipos de salud en el colegio?
31. ¿Qué cambios ha significado la entrada de los equipos de salud en la dinámica del colegio?
32. ¿Cómo cree usted que perciben los(as) adolescentes la presencia de los equipos de salud en los colegio?
33. ¿Cómo cree usted que perciben los(as) profesores(as) la presencia de los equipos de salud en los colegio?
34. ¿Cuáles percibe ustedes han sido los mayores beneficios de la entrada de los equipos de salud en los colegios para los(as) alumnos(as)?
35. ¿Cuáles percibe ustedes han sido los mayores beneficios de la entrada de los equipos de salud en los colegios para los(as) profesores(as)?
36. ¿Cuáles percibe han sido las mayores dificultades en la entrada de los equipos de salud en los colegios para los(as) alumnos(as)?
37. ¿Cuáles percibe han sido las mayores dificultades en la entrada de los equipos de salud en los colegios para los(as) profesores(as)?
38. ¿Identifica resistencias por parte de profesores(as) en la entrada de los equipos de salud al colegio?
39. ¿Existen actividades docentes conjuntas entre profesores(as) y equipos de salud? ¿Cuáles?
40. ¿Cómo ha sido esta experiencia?
41. ¿Qué han aportado los equipos de salud a los profesores(as) del colegio? Profundizar en lo relativo al componente afectividad y sexualidad.
42. ¿Qué han aportado los profesores(as) a los equipos de salud?
43. ¿Cuál cree que ha sido el impacto en la salud sexual y reproductiva de los(as) alumnos la instalación de los equipos de salud en el colegio?
44. ¿Cuál cree que ha sido el impacto en el embarazo adolescente la instalación de los equipos de salud en el colegio?

GUÍA DE ENTREVISTA
Adolescentes y Jóvenes

ESTUDIO ETNOGRÁFICO PARA LA PROMOCIÓN DE LA SALUD SEXUAL/REPRODUCTIVA Y LA PREVENCIÓN DEL EMBARAZO ADOLESCENTE EN CHILE: IDENTIFICANDO DESAFÍOS Y PROPONIENDO SOLUCIONES.

Investigadora Principal: Alexandra Obach, PhD

V. Preguntas generales acerca de la implementación del Programa 3A en la comuna de Lo Prado:

45. ¿Conoces el Programa 3A?
46. ¿Cómo lo definirías?
47. ¿En qué actividades del Programa 3A has participado?
48. ¿Qué te han parecido esas actividades?
49. ¿Qué aspectos del Programa 3A son los que más valoras?
50. ¿Qué aspectos del Programa 3A son los que menos valoras?
51. ¿Qué te ha aportado el Programa 3A? Indagar en ámbitos de conocimientos, ámbitos de socialización, ámbitos emocionales, salud mental.
52. ¿Cuáles consideras son los aspectos más novedosos de este programa?
53. ¿Cómo describiría las salas de salud o salas de bienestar?
54. ¿Utilizas estas salas? Razones por qué si/no. ¿Para qué? En qué horarios?
55. ¿Qué opinión tienes del componente de afectividad y sexualidad del Programa 3A?
56. ¿Qué actividades contempla este componente?
57. ¿Qué has aprendido sobre este tema a través del programa?
58. ¿Qué mejorarías del programa y del componente de afectividad y sexualidad específicamente?

VI. Preguntas exclusivas sobre equipos de salud:

59. ¿Conoces el Espacio Amigable del consultorio? ¿Lo utilizas?
60. ¿En qué se diferencia la atención de salud sexual y reproductiva a adolescentes y jóvenes que se entrega en los centros de salud y la que se entrega en el colegio a través del Programa 3A?
61. ¿Cómo ha sido tu experiencia de tener a equipos de salud a interior del colegio?
62. ¿Marca esto una diferencia con respecto a otros colegios?
63. ¿Cómo es el vínculo de los alumnos con los equipos de salud?
64. ¿Qué relación establecen?
65. ¿Para qué buscan los alumnos a los equipos de salud?
66. ¿Son accesibles los profesionales de salud al interior del colegio?
67. ¿Consideras que los profesionales de salud saben sobre los temas de sexualidad que a ti te interesan?
68. ¿Están los equipos de salud disponibles para hablar de temas de sexualidad con los alumnos?
69. ¿Qué piensas del embarazo en la adolescencia?
70. ¿Tener a equipos de salud en el colegio ayuda a prevenirlos? ¿De qué manera?
71. ¿Qué piensas de las ITS?
72. ¿Tener a equipos de salud en el colegio ayuda a prevenirlas? ¿De qué manera?
73. ¿Los profesionales de salud que están en los colegios manejan temas de diversidad sexual?
74. ¿Cuál es la fortaleza de tener a profesionales de salud en el colegio?
75. ¿Cuál es la debilidad de tener a equipos de salud en el colegio?

VII. Preguntas exclusivas sobre profesores:

76. ¿Qué información manejan los profesores sobre salud sexual y reproductiva?
77. ¿Cómo es la educación sexual que imparten los profesores en el colegio?
78. ¿Están los profesores disponibles para hablar de temas de sexualidad con los alumnos?
79. ¿Qué tipo de vínculo mantienen los alumnos con los profesores?
80. ¿En qué se diferencia el vínculo que mantiene los alumnos con los profesores y con los profesionales de la salud?
81. ¿Están alineados profesores y equipos de salud en su quehacer en el colegio?
82. ¿Identificas resistencias por parte de profesores(as) en la entrada de los equipos de salud al colegio?
83. ¿Existen actividades docentes conjuntas entre profesores(as) y equipos de salud? ¿Cuáles?
84. ¿Cómo ha sido esta experiencia?
85. ¿Qué han aportado los equipos de salud a los profesores(as) del colegio? Profundizar en lo relativo al componente afectividad y sexualidad.
86. ¿Qué han aportado los profesores(as) a los equipos de salud?
87. ¿Cuál cree que ha sido el impacto en la salud sexual y reproductiva de los(as) alumnos la instalación de los equipos de salud en el colegio?
88. ¿Cuál cree que ha sido el impacto en el embarazo adolescente la instalación de los equipos de salud en el colegio?
